# Physical model test on soil slopes under the effect of bedrock fault dislocations

**DOI:** 10.1371/journal.pone.0331840

**Published:** 2025-09-05

**Authors:** Yijie Song, Jianyi Zhang, Jianke Ma, Xiaobo Li, Haonan Zhang, Ruijie Hu

**Affiliations:** 1 School of Geological Engineering, Institute of Disaster Prevention, Langfang 065201, China; 2 Hebei Key Laboratory of Earthquake Disaster Prevention and Risk Assessment, Langfang 065201, China; 3 Key Laboratory of Building Collapse Mechanism and Disaster Prevention, China Earthquake Administration, Sanhe, China; 4 Key Laboratory of Earthquake Engineering and Engineering Vibration, Institute of Engineering Mechanics, China Earthquake Administration, Harbin, China; Southwest Petroleum University, CHINA

## Abstract

Bedrock fault dislocations significantly influence the rupture instability of rock and soil slopes adjacent to fault zones. Understanding the dynamic processes, kinematic characteristics, and genesis mechanisms of landslides induced by strong seismic fault dislocations is crucial for advancing the theoretical framework of landslide studies. This paper presents a representative experiment simulating the emergence of seismic faults (internal rupture belts within the soil mass) at the shoulders and toes of slopes due to bedrock fault dislocations. Two failure modes were examined: soil mass rupture at the shoulders and the toe of the slope. The rupture at the shoulders was found to pose a greater threat to the stability of the soil slopes under intense fault dislocations. Furthermore, the genesis mechanisms and dynamic characteristics of sandy soil slopes destabilized by reverse fault dislocations can be categorized into three stages: Stage I, the gestation stage of slope soil mass rupture; Stage II, the development stage of the slope soil mass rupture zone; and Stage III, the stage of rupture-induced landslides. The study findings can be useful in slope stability analysis under bedrock dislocation conditions as well as for slope protection and management near fault zones. Both model tests and field cases of earthquake damage demonstrate a strong correlation between the location of the main fracture zone on the slope surface (at the slope shoulder and slope toe) and the displacement of the two walls of the bedrock fault.

## Introduction

Previous earthquakes have demonstrated that landslides are influenced by faults, with more than half of landslides occurring within approximately 5–10 km on both sides of the surface rupture zone caused by seismogenic faults. Landslide disasters caused by bedrock fault displacement have led to substantial economic losses and casualties [1–10]. Therefore, conducting a thorough investigation into how fault displacements impact slope stability, as well as determining the governing laws of instability and failure, has become crucial in slope engineering research.

Landslides triggered by seismic fault dislocations have been extensively studied. Xu and Li [6] conducted on-site mapping and research on hundreds of large-scale landslide disasters triggered by the main fault during the 2008 Ms 8.0 Wenchuan earthquake. Their findings indicated that most of the landslides occurred on the hanging walls of active faults, exhibiting a clear “hanging wall effect.” The primary triggering source for these large-scale landslides was the direct seismic force caused by sudden fault dislocations. Shao [9] statistically investigated landslides caused by the 2018 Ms 7.4 Palu earthquake, noting that most small-to-medium landslides exhibit seismic fault-control, concentrated near mountain ridges with significant terrain effects. Moreover, they observed that the majority of landslides occurred on upper slope sections. In their survey of the 2023 Mw 7.8 Türkiye earthquake, Yan [11] identified dolomite slope collapses induced by faults, which resulted in the Islamiye and Tepehan landslides. Through satellite imagery analysis, Pánek [1] determined that mudstone landslides in the southeastern Greater Caucasus are predominantly concentrated in high-altitude terrain, often developing on the uphill sides of thrust faults that can induce large-scale slope deformations such as trenches and tensile cracks. Peng [12] investigated Liujiabao Village landslides in Tianshui City, studying the evolutionary process of loess-mudstone landslides controlled by faults. Four distinct stages were identified: embryonic, creep cracking, sliding, and stabilization. Huang [13] investigated rarely documented large-scale landslides that have occurred in China since the 20th century, classifying their failure mechanisms into a “three-stage model” involving sliding, tensile cracking, and shearing, as well as the creep shearing mode of inclined rock layers.

Most existing studies primarily focus on field investigations of seismic damage in fault-triggered landslides, yet rarely examine the deformation and failure mechanisms of slopes undergoing bedrock fault dislocation. Given that slope monitoring methods documented in these studies predominantly rely on conventional techniques such as displacement measurements, they can only detect large-scale deformations or macro-instability post-occurrence. These traditional monitoring techniques are inadequate for capturing the evolution processes and mechanisms of micro-cracks and micro-fractures within geotechnical masses—specifically, internal fracturing changes preceding slope failure.

Recently, microseismic monitoring technology has emerged as an advanced 3D spatial monitoring technique for micro-fracturing in geomaterials (rock and soil masses), with significant advancements. When geomaterials undergo localized elastoplastic concentration due to external factors, the accumulated energy reaches a critical threshold, triggering the generation and expansion of microcracks along with the release of stress waves (termed microseismic waves) that rapidly propagate through surrounding media.

Through the integration of microseismic monitoring technology into fault slope stability analysis, Xu [14] pioneered novel analytical methods for such studies. Similarly, Zhang [15] and Yang [16] utilized microseismic systems to monitor slopes, successfully identified deep-seated rock deformation zones and mapping unknown geological structures induced by microseismic activity on fault-rich rocky slopes, thereby enhancing slope construction assessments. Li [17] developed a large-scale vibration testing apparatus coupled with a microseismic monitoring system to investigate vibration-induced failure characteristics in coal-rock fracture sites. By incorporating vibration mechanics principles, they elucidated the resonance amplification mechanism, detected discernible microseismic signals during incipient rupture of coal-rock specimens that attenuated proportionally to propagation distance. Employing a dynamic acoustic-electric monitoring system for coal rocks, Jing [18] analyzed microseismic signals generated during coal seam pressure relief blasting at multiple locations, revealing frequency spectrum evolution characteristics and demonstrating exponential energy decay relative to propagation distance.

Meanwhile, researchers have conducted shaking table model tests to investigate slope stability within fracture zones generated by bedrock dislocation. Huang [19] demonstrated that fault zones within slopes significantly amplify seismic acceleration, thus exacerbating slope damage. Through cross-validation of physical model tests and field case studies, Thierry Nalpas et al [20] revealed that fault zones induce the most severe damage at the slope shoulder. By monitoring soil slopes with microseismic systems, Yin [21] classified landslide failure modes into three categories: sparse cracking, unstable primary cracking, and retrogressive failure characterized by dense secondary cracking. Xiao [22] performed geomechanical model tests on landslides using microseismic monitoring as the principal measurement technique, revealing a torsional-rupture failure mechanism. Fang et al. [23] utilized an integrated multi-parameter monitoring system coupling displacement, acoustic emission, and earth pressure measurements. Through physical slope modeling, they documented the full progression from progressive deformation to catastrophic failure in excavated slopes. In complementary research, simplified landslide models were established to analyze deformation patterns, failure characteristics, and dynamic responses of landslides while quantifying slope acceleration [24–28]. Through experimental modeling of thrust fault impacts on rock slopes, Tokashiki [29] identified active failure mechanisms comprising shear, flexural, and planar sliding. Li [30] developed a 3D discrete-element model and conducted numerical simulations of riverbank slopes controlled by fault zones, determining the fault zone’s weak interlayer as the critical geological structure controlling failure. Rong and Wang [31] simulated multi-fault rupture zone effects on steep limestone slopes using FLAC3D, identifying displacement concentration proximal to rupture zones that significantly heightened slope vulnerability. Validated through field experiments, the three-dimensional Discontinuous Deformation Analysis (DDA) method has enabled high-fidelity simulation of rock slope failure processes based on recent studies [32–33]. This approach accurately reveals the evolutionary patterns of rockfall motion and the cascade failure mechanism in columnar rock masses, delivering crucial numerical validation for physical modeling. Collectively, these studies have substantially advanced mechanistic understanding of slope deformation and instability mechanisms under bedrock fault displacement.

In summary, slope seismic damage within primary fault zones during strong earthquakes is dominated by seismic faults or surface rupture belts. Research on bedrock-dislocation-induced landslides has primarily focused on the kinetic response of slope rupture zones following bedrock displacement, yielding significant advances. However, the evolutionary processes and characteristics of internal rupture zones during bedrock dislocation—specifically: (1) rupture dynamic propagation induced by abrupt bedrock dislocation in soil slopes, (2) real-time internal geomechanical responses, and (3) ultimate failure modes—remain unresolved core scientific challenges. These knowledge gaps stem from critical constraints: field investigations face limitations in capturing and authentically reconstructing instantaneous rupture dynamics and micromechanisms due to operational restrictions preventing trenching of internal rupture zones and frequent lack of instrumentation; existing physical modeling typically introduces pre-embedded weak discontinuities within slopes or overburdens to simulate vibration damage under seismic loading, yet fails to physically replicate the driving process of bedrock dislocation itself and its rupture-inducing effects on overlying soil slopes; numerical simulations are constrained by insufficient geotechnical parameters and boundary conditions from available experiments, and more fundamentally, computationally viable constitutive models and numerical algorithms required to accurately simulate large soil deformations and rupture propagation induced by bedrock dislocation generally prove elusive, compromising result reliability and generalizability.

To overcome these limitations—particularly in capturing real-time microscopic characteristics and internal macroscopic failure phenomena of bedrock-dislocation-driven slope failures, critical information unobtainable through conventional methods—this study proposes and designs a novel large-scale 1g physical modeling apparatus. This experimental system directly simulates bedrock dislocation loading along seismogenic faults, specifically investigating how dislocation magnitude and location govern rupture propagation paths, failure severity, and ultimate instability modes in sandy slopes. The apparatus integrates a multi-sensor system comprising a microseismic monitoring array for real-time internal micro-fracture detection, supplemented by dynamic earth pressure cells, high-precision displacement transducers, and high-definition cameras. Utilizing this unique research platform, the study:1) Establishes generalized slope models for sandy sites under bedrock dislocation conditions; 2) Comprehensively analyzes internal dynamic responses (microseismic activity, dynamic earth pressures, displacement field evolution) and surface crack development patterns during dislocation;3) Reveals failure mechanisms and establishes kinematic failure modes of soil slopes under varying conditions.

These findings provide physically-based evidence and novel insights for slope stability analysis under bedrock dislocation, while delivering validated technical foundations for disaster prevention and mitigation designs of critical engineered slopes traversing/near fault zones.

## Site model test on soil slope under reverse bedrock fault

### Model test device

**Fig 1:**
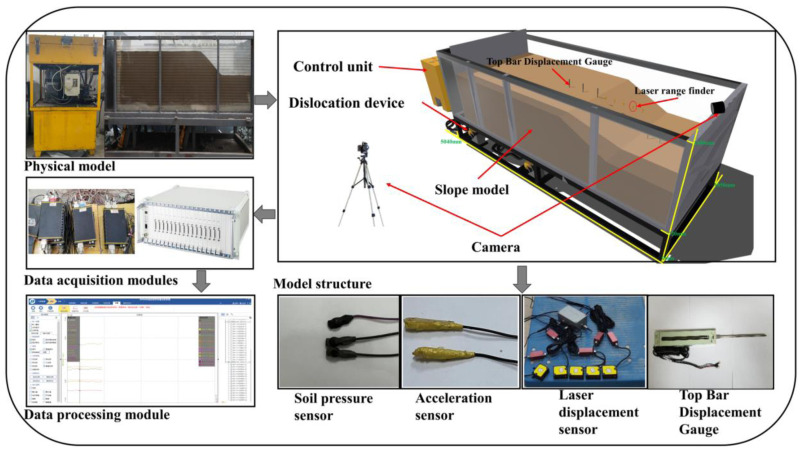
Experimental setup for the soil slope model under bedrock dislocation caused by seismic rupture.

The modeling apparatus (Fig 1), comprises of a bedrock dislocation platform (composed of a soil box, reaction support base, hydraulic actuator, and fault dip adjustment system), a connection system (including hydraulic oil pipes and displacement sensor lines), and a hydraulic loading system designed to simulate bedrock dislocation inputs. The front and back panels of the soil box are constructed from 0.025-m-thick high-strength organic glass, each reinforced with three external vertical stiffening ribs to ensure structural stability. The side panels are fabricated from 0.015-m-thick high-strength steel plates. The base features a double-layered steel plate structure embedded with L-section steel beams, comprising movable and fixed steel plates to simulate differential displacements of upper and lower bedrock layers beneath overburdened soil. A flexible canvas enclosure surrounds the steel plates and box periphery, preventing soil leakage during cyclic actuator operation. The hydraulic actuators are positioned beneath the L-shaped movable steel plate, supported by steel angle brackets on the base. Adjustments to these brackets enable variation of loading direction and angle, thereby simulating “overburden soil–soil slope” failure modes under diverse fault types, bedrock displacement angles, and test conditions.

The device’s modularity permits versatile test configurations through adjustable slope positioning. By controlling actuator loading directions and support angles, it replicates soil slope failures under varying fault kinematics, dip angles, displacement magnitudes, and spatial relationships. This configuration enables systematic investigation of slope instability under multifactorial conditions. Detailed sensor specifications are provided in Table 1.

**Table 1:** Summary of sensor specifications.

No.	Type	Product Code	Specifications
**1**	Top Bar Displacement Gauge	YHD-200	Range: ± 100 mm Bridge configuration: Full-bridge or half bridge Gauge factor: 1.005 Sampling frequency: 0–50 Hz
**2**	Laser displacement sensor	FTJP-1	Range: 10–1000 mm Accuracy: 0.1 mm Sampling frequency: 200–5000 Hz
**3**	Soil pressure sensor	ESP-II	Range: ± 50 kPa Accuracy: 0.5% FS Measurement type: Voltage Sampling frequency: 0–100 Hz
**4**	Acceleration sensor	DCIEM-M	Range: ± 1 g Sensitivity: 0.1% Measurement type: Voltage Sampling frequency: 0–100 Hz

**Fig 2:**
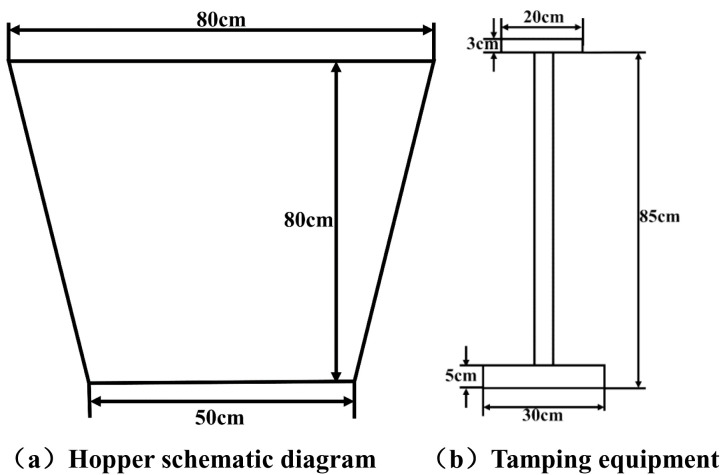
Design schematic diagram of hopper and ramming device. (a) Hopper schematic diagram. (b) Tamping equipment.

Prior to each test, rod extensometers were calibrated by applying preset stepped displacements (0–20 mm) while simultaneously verifying physical scale readings against data acquisition system outputs. Laser displacement sensors underwent translation-method calibration using a planar calibration plate positioned coplanar with the laser emission plane; outputs were recorded during controlled uniaxial translation and calibrated against known plate positions. Earth pressure cells were calibrated using soil-column (0–50 kPa) and water-column methods through the following procedure (Fig 2): after placing 15 cm of sand in a hopper compacted to 10 cm height, the pressure cell was positioned on the surface (ensuring absence of rigid inclusions), connected to the data acquisition system, and activated. Successive 15 cm sand layers were added and compacted to 10 cm with continuous data recording until 50 cm depth, with measured pressures calibrated against theoretical overburden stresses. Microseismic sensors were dynamically calibrated against nationally certified 941B vibrometers by co-locating both sensors on a miniature frame, exciting the frame while recording data, and calibrating through signal comparison with the reference vibrometer.

### Experimental scheme design

**Fig 3:**
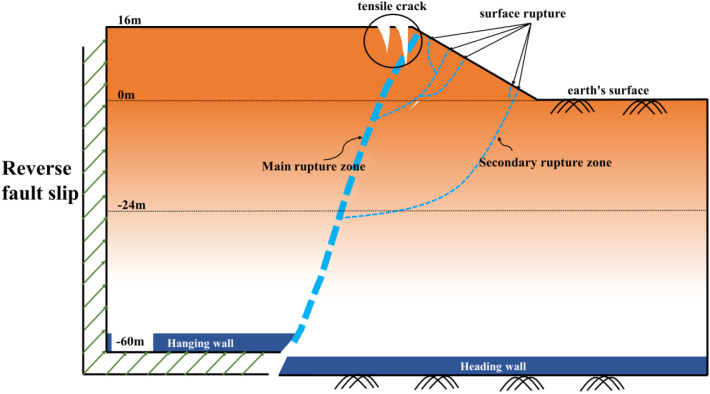
Slope shoulder failure conceptual diagram.

**Fig 4:**
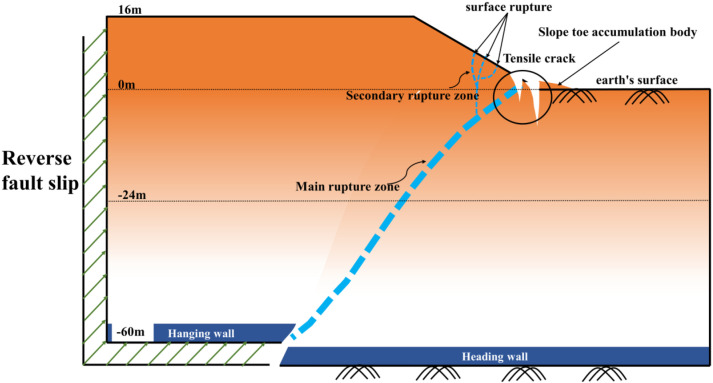
Slope toe failure conceptual diagram.

To directly elucidate the governing mechanisms through which bedrock dislocation magnitude and location control rupture propagation pathways, failure severity, and ultimate instability modes in sandy slopes, this study establishes a generalized overburden slope model based on bedrock fault dislocation hazards (Figs 3 and 4), developed from prior constant-gravity physical modeling apparatuses [34–36]. This conceptual model directly simulates the bedrock dislocation driving process while integrating multi-sensor instrumentation—specifically microseismic arrays, earth pressure cells, and displacement transducers—to capture real-time rupture propagation dynamics, internal geomechanical responses, and instability mode evolution in soil slopes under controlled dislocation parameters. The system ultimately constitutes a physical model of bedrock-dislocation-induced hazards, faithfully replicating the complete rupture zone development process driven by bedrock dislocation.

In the generalized slope model of this study, the overburden thickness exceeds the 60m threshold specified in the Chinese National Standard GB 50011−2010 (Code for Seismic Design of Buildings) [37] where fault dislocation effects become negligible for seismic fortification intensity level 8. The prototype slope and overburden thickness were set at 40m with a slope angle of 30°. Model boundaries were established following Zhang [38]et al.‘s research: distance from toe to left boundary = 1.5 H; distance from crest to right boundary = 2.5 H; total vertical height = 2 H. Based on these scaling relationships and prototype parameters, the final model dimensions were established as: slope and overburden thickness = 1m, slope height = 0.4m, and slope length = 0.7m (corresponding to a prototype dimension of 40m through geometric scaling).

For bedrock dislocation magnitude, Huang [39] established an empirical relationship between surface wave magnitude (M_S_) and fault dislocation displacement (D_V_) through regression analysis of historical earthquake fault displacement data from mainland China:


$$LgD_{V}=a+bM_{S}$$ (1)

The regression coefficients are a = −2.61 and b = 0.38. For extreme seismic scenarios with Ms ≈ 8.5, this model predicts a D_V_ of ~ 4.17 m. Therefore, this study adopts a design fault displacement of 4 m, which scales to 100 mm in the physical model through dimensional similitude, effectively simulating bedrock fault displacement effects on slope failure mechanisms and overburden rupture patterns under M_S_ ≈ 8.5 seismic conditions.

A bedrock dislocation rate of 2 mm/s was maintained. Research on strong earthquake surface rupture formation [40] indicates fault dislocation rates typically range from 0.008 to 6.2 mm/s, placing this test rate within the median range to effectively simulate stick-slip behavior. Critical condition studies for overburden rupture [41,42] confirm that when bedrock dislocation reaches 3%−5% of soil layer thickness (corresponding to 1–2 m dislocation for 40 m overburden), rupture propagation requires 40–60 s duration. At 2 mm/s, the model achieves 100 mm dislocation in 50 s, conforming to measured rupture durations within engineering tolerance. This quasi-static rate enables clearer observation of progressive rupture development.

Fault dip angle selection followed Huang’s [39] statistical analysis of near-surface fault systems in mainland China, which revealed that high-angle reverse faults (60°-80°dip) constituted 72.5% of sampled systems, with approximately 70°dip angles demonstrating the greatest engineering prevalence. Consequently, 70°was selected for this investigation.

**Fig 5:**
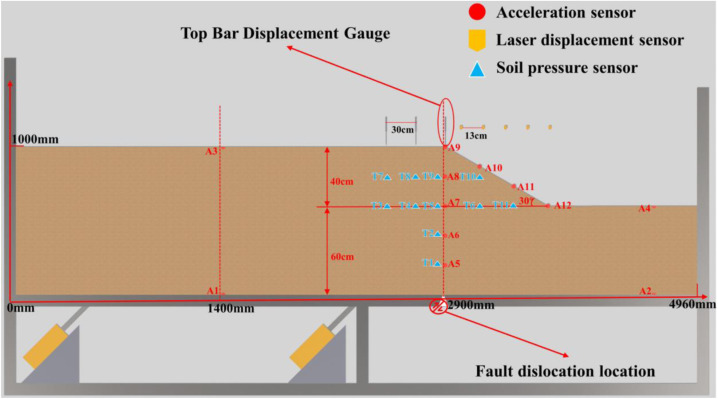
Layout of soil slope (Sand Soil) shoulder rupture.

**Fig 6:**
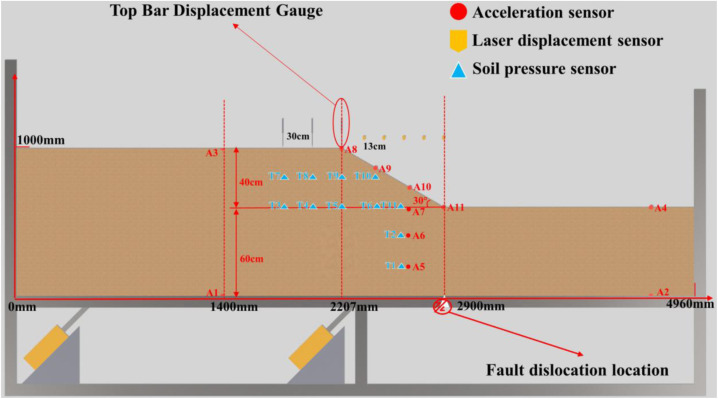
Layout of soil slope (Sand Soil) toe rupture.

This experiment integrated the generalized slope model under bedrock fault control, deploying earth pressure cells, displacement transducers, and microseismic sensors at optimized positions. Benchmark tests were designed to induce characteristic failure modes at slope shoulders and toes in sandy slopes subjected to bedrock fault dislocation, as detailed in Table 2 and Figs 5–6.

**Table 2:** Parameter values for soil slope site test.

Test.	Total dislocation amount (mm)	Single dislocation amount (mm)	Shoulder position (mm)
①	100	10	2900
②	100	10	2207

To mitigate wave refraction and reflection at model container boundaries, a specialized framework (Fig 1) was fabricated using high-strength acrylic panels for front and rear surfaces, with external steel plates forming rigid fixed boundaries. Energy-absorbing foam panels were installed between steel plates and soil to minimize boundary reflections, rigorously adhering to Lin & Wang’s [43] criterion requiring the minimum distance from slope shoulder to boundary (2.2 m) exceeded twice the horizontal projection of slope length (0.7 m).

Microseismic sensor positioning errors were minimized via optimized array configurations developed during calibration trials (final layout in Figs 5–6), identifying the central longitudinal section as the optimal instrumentation plane. All microseismic accelerometers, earth pressure cells, rod extensometers, and laser displacement sensors were concentrated along this profile to maximize localization accuracy for internal fault activity and micro-crack nucleation.

Instrumentation layouts (Figs 5–6) comprise:12 microseismic accelerometers monitoring acceleration responses at rupture locations;11 earth pressure cells tracking stress variations at critical slope positions; Five rod extensometers measuring surface deformations; Five laser displacement sensors quantifying displacement fields.

### Preparation of sandy slope model

The theoretical foundation for similarity design in physical modeling is governed by dimensional analysis principles differentiating qualitative and quantitative approaches based on material similarity.

This study employed a 1:40 geometrically scaled model developed using a multifunctional bedrock dislocation platform. The qualitative modeling approach-maintained prototype bulk density (Table 3) to simulate soil/rock mass rupture and slope deformation mechanisms. Through similarity theory with fundamental quantities ρ (density)、 L (length), and g(gravity), similarity constants were derived considering prototype dimensions, container capacity, boundary effects, and site constraints: Cρ = 1、 CL = 40、 Cg = 1. This scaling ratio has been extensively validated in bedrock dislocation simulations, fracture propagation studies, and analogous geotechnical problems [44–47], demonstrating reliability within define engineering contexts.

**Table 3:** Soil parameters and their similarity constants in the model.

Physical quantity	The constant of similarity of soil
L: Length	$C_{L}=40$
ρ: Density	$C_{\rho }=1$
g: Gravity acceleration	$C_{g}=1$
τ: Soil pressure	$C_{\tau }=C_{L}\cdot C_{\rho }\cdot C_{g}=40$
D_L_: Rod displacement	$D_{L}=40$
μ: Poisson ratio	$C_{\mu }=1$
ε: Strain	$C_{\varepsilon }=1$
u: Displacement	$C_{\varepsilon }=C_{L}=1$

**Fig 7:**
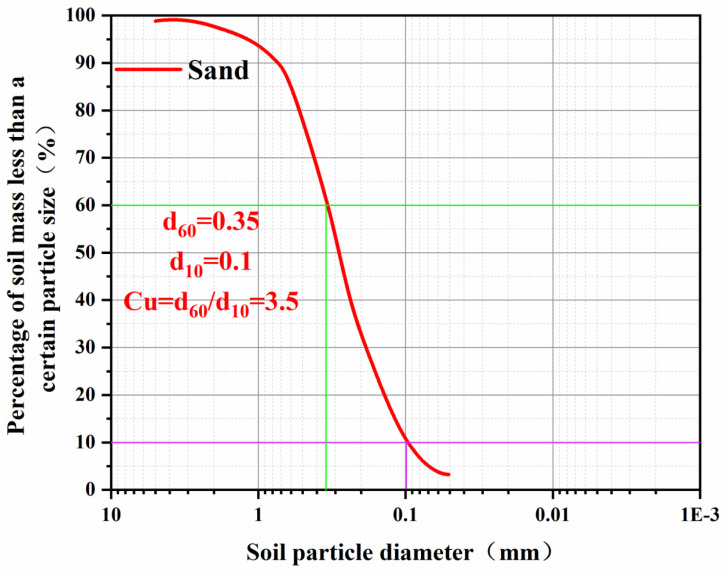
Particle size distribution curve of sand soil.

Fig 7 shows the particle size distribution curve of the sandy soil used in the experiment, which had a cohesive (C) of 0 kPa, an internal friction angle (φ) of 27.6°and a density is 1.675 g/cm^3^ (Table 4) .

**Table 4:** The basic physical parameters of sand soil.

d60	d10	Cu	C	φ
0.35	0.1	3.5	0	27.6°

Following preliminary trials, the experimental soil layer was prepared according to the principle of quantitative placement, layered compaction, interface leveling, and deficiency compensation. The procedure comprised:

1. Model Container Preparation

The fully assembled model container had its actuator angle fixed at 70°. External adhesive marker tapes were applied at 50-mm intervals to guide soil leveling. After inspection, a plastic membrane was installed at the base-container interface to prevent soil leakage during the filling. Soil placement commenced upon completion.

2. Layered Compaction & Sensor Installation

Soil was placed in 150 mm lifts, each compacted to 100-mm thickness. During compaction, soil pressure sensors were embedded per the predefined layout. Upon reaching 1000 mm compacted height, a 40 cm-high slope with 30° inclination was formed. Surface instrumentation comprised rod extensometers and a laser displacement sensor, supplemented by cameras positioned above and facing the container.

Model preparation employed layered compaction to control soil variability: sand was initially placed at 150 mm loose-lift thickness and compacted to 100 mm design thickness. Following each layer compaction, cutting ring sampling was conducted via grid-point method (≥6 sampling points per layer with duplicate tests per point) to simultaneously determine density, water content, and degree of compaction. Results underwent cross-verification ensuring soil uniformity and repeatability. Additionally, moistened geotextile covered compacted surfaces prior to subsequent layer placement to maintain moisture content and prevent excessive evaporation.

3. Data Acquisition & Recording

The hydraulic actuator was controlled to incrementally displace by 10 mm per substage, with data recorded at each interval. Ten substages constituted one complete test cycle. During each substage, sensor data were systematically collected using a data acquisition system. High-speed cameras recorded the full progression to ensure comprehensive recording of failure progression.

## Failure characteristics of sandy soil slope with two bedrock dislocations

### Analysis of slope site damage

**Fig 8:**
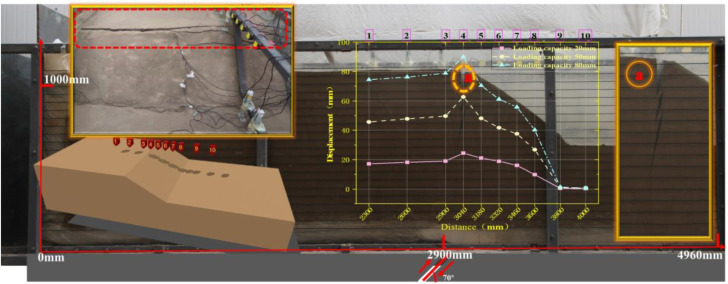
Site map of soil slope shoulder rupture in Experiment 1.

**Fig 9:**
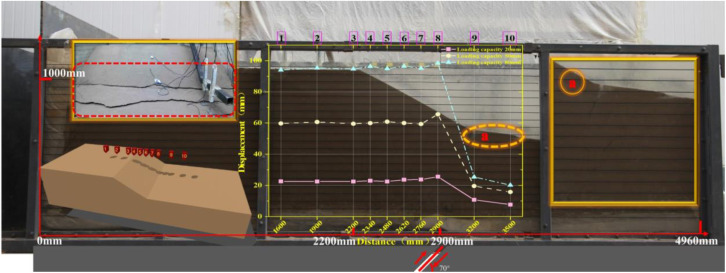
Site map of soil slope toe rupture in Experiment 2.

**Fig 10:**
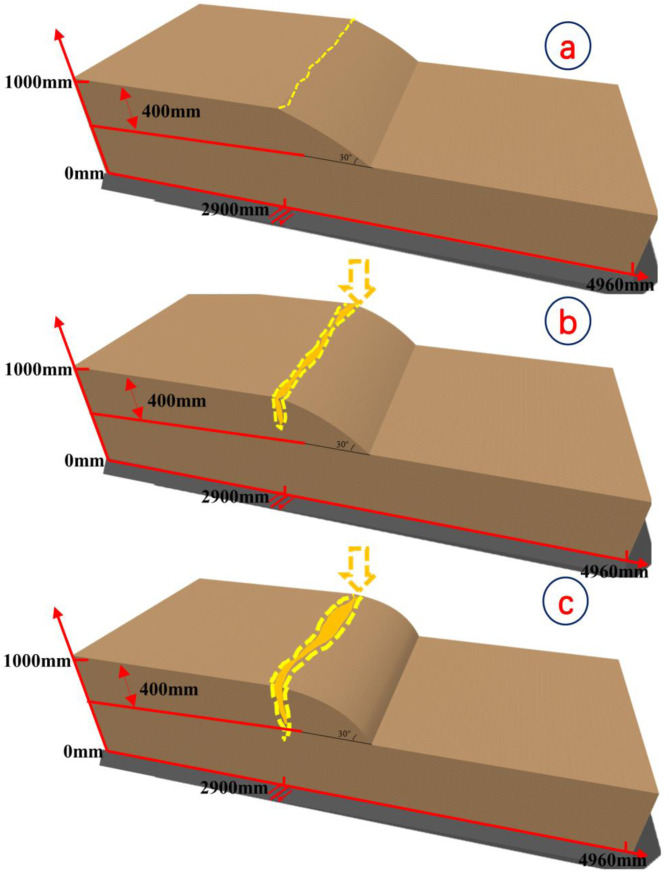
Stage evolution diagram of soil slope shoulder rupture (Experiment 1).

**Fig 11:**
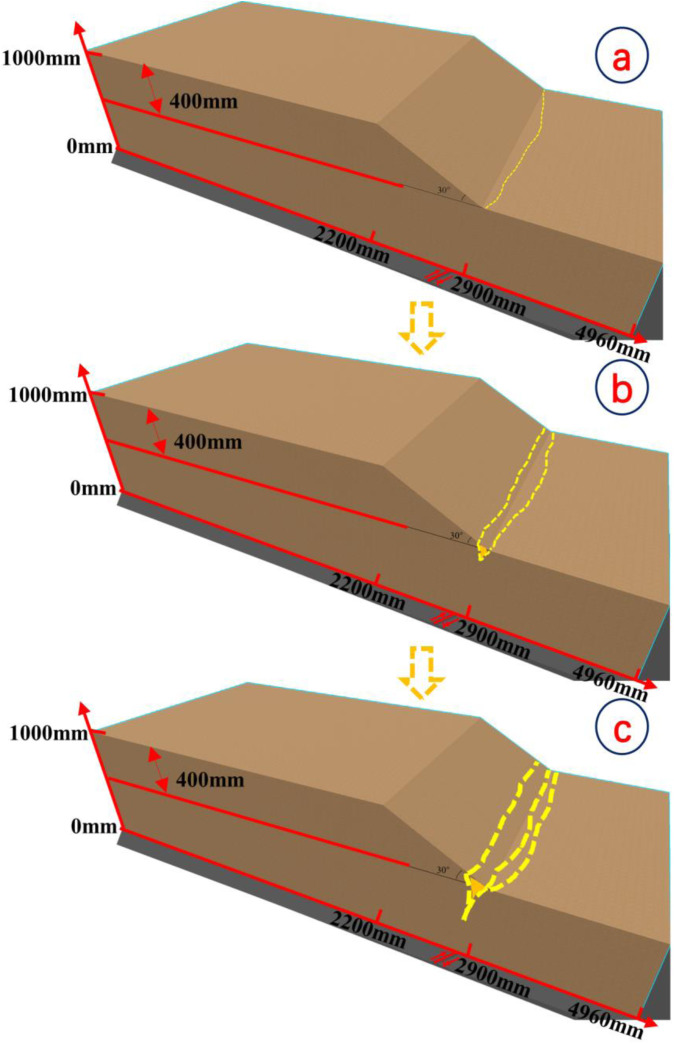
Stage evolution diagram of soil slope angle rupture (Experiment 2).

Figs 8 and 9 illustrate the on-site damage process of the slope and the overburden. Figs 10 and 11 present the generalized stage diagrams of slope rupture at the experimental site. In Experiment 1, when the bedrock fault dislocation reached 20 mm, passive tensile cracks appeared on the slope shoulder (Fig 8), whereas Experiment 2 generated active compression-shear cracks at the toe of the slope (Fig 9). The tensile cracks in Experiment 1 gradually propagated into banded patterns (Fig 8), while the compression-shear cracks in Experiment 2 extended downward along the slope surface (Fig 9). When the bedrock fault dislocation reached 50 mm, Experiment 1 developed a wide downward-spreading vertical rupture zone, with subtle tensile cracks near the slope shoulder on the surface (Fig 10b). In Experiment 2, another downward shear crack appeared at the toe, gradually expanding. Simultaneously, due to the obstruction of an obstructing platform at the toe incipient debris accumulation formed (Fig 11b). At a dislocation of 80 mm, Experiment 1 formed a conical tensile rupture zone that continued to develop downward, penetrating the slope body. The tensile cracks on the slope shoulder and surface further widened (Fig 10c). In Experiment 2, a third shear crack emerged at the toe, causing partial slope causing material expulsion onto the platform and scattered at the toe and front edge, forming slope-toe accumulation. As shear cracks continued to develop and expand, a narrow, short, short inclined rupture zone appeared (Fig 11c). Both experiments produced rupture zones, with Experiment 1 exhibiting the widest bud-like shear rupture zone and the most severe slope instability. This indicates that under similar soil properties, slope failure in shoulder rupture zones will be more severe and prone to instability with increasing bedrock fault dislocation.

Under reverse fault dislocation (20–80 mm), the slope surface in Experiment 1 exhibited significant ‘bulging’ toward the free face from rupture initiation at the shoulder (Fig 8), whereas Experiment 2 primarily showed ‘translational’ motion from reverse-fault thrust (Fig 9).

**Fig 12:**
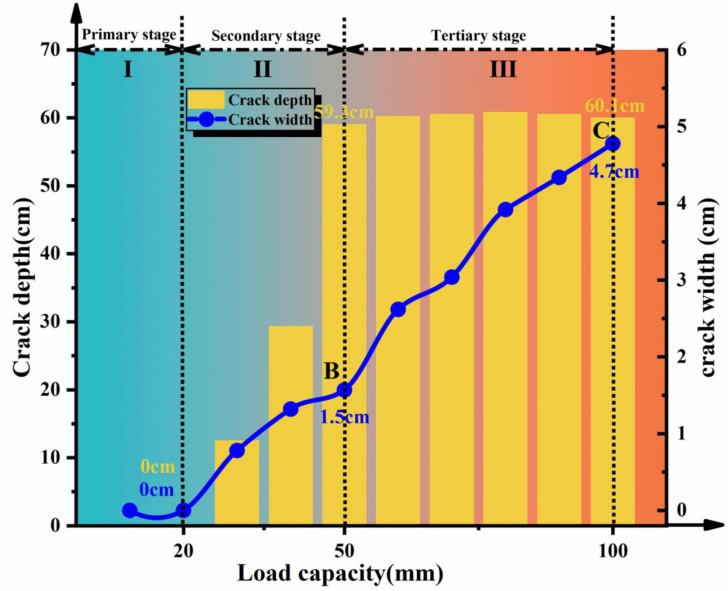
Statistical chart of depth and width of rupture zone during shoulder failure.

**Fig 13:**
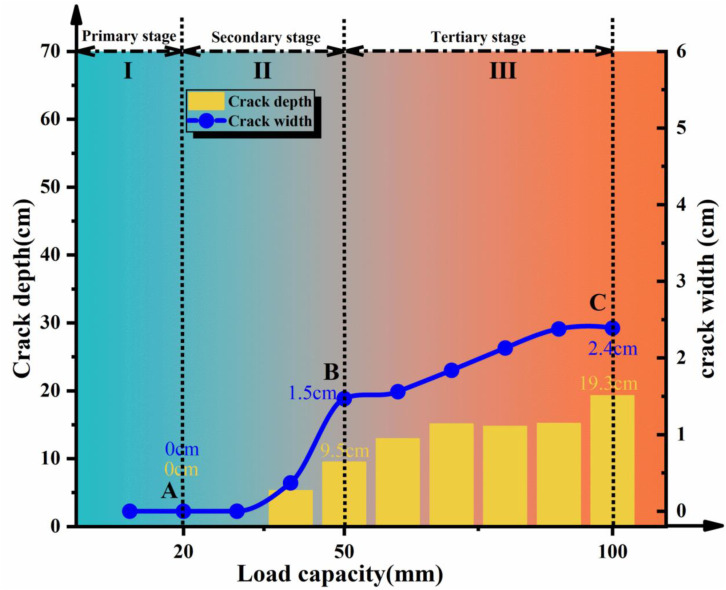
Statistical chart of depth and width of the rupture zone during slope toe failure.

From the results shown in Figs 10 and 11 and Figs 12 and 13, the dynamic evolution process of slope instability can be categorized into three stages. During the first stage, with an increase in bedrock dislocation, subtle cracks appeared in the failure locations or failure zones, accompanied by slight changes in the crack width. These initial cracks indicate or denote of slope instability, marking the beginning of rupture development and changes in soil behavior.

As the dislocations continued to accumulate, the process advanced to the second stage, characterized by rapid and extensive changes in the crack depth and width, indicating intensified slope instability. Cracks in both failure models transitioned from localized to widespread propagation and deepen throughout the entire slope. Ultimately, upon reaching the dislocation threshold, the models entered the third stage. Here, the width and depth of the main crack stabilized in the toe failure model, while the depth of the main crack in the shoulder failure model. However, its width expanded rapidly, indicating complete slope instability and a tendency to slide.

Notably, when the rupture zone caused by bedrock faults is situated at the slope shoulder, it presents higher hazard potential secondary landslides compared to when it occurs at the toe of the slope. This heightened risk is due to amplified kinematic impacts of fault rupture zones on the slope shoulder, potentially leading to larger-scale deformation and surface damage, intensifying instability.

### Microseismic response analysis of slopes

**Fig 14:**
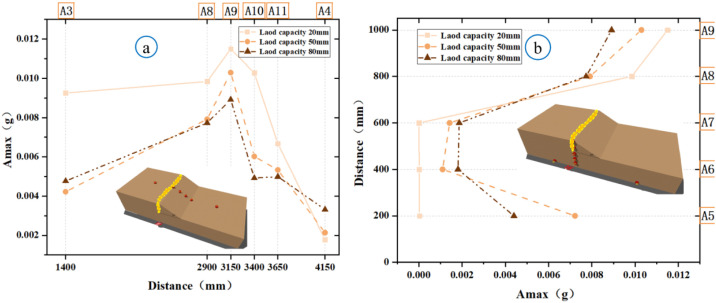
Comparison of microseismic peak values during slope shoulder soil failure (Experiment 1).

**Fig 15:**
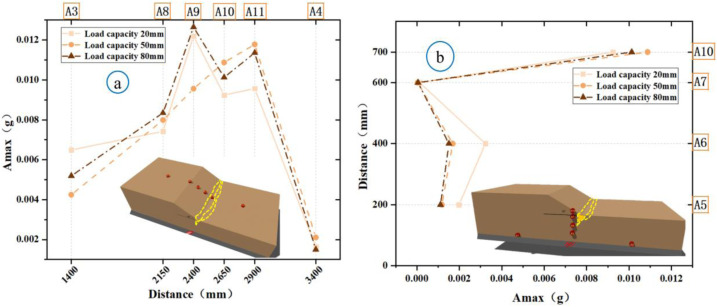
Comparison of microseismic peak values during soil failure at the toe of the slope (Experiment 2).

Microseismic sensors capture real-time elastic waves generated during the rupture of rocks and soil, providing critical insights into the evolution of such events. To investigate how the displacement of bedrock faults influences both the surface and interior of soil slopes at different locations, the peak horizontal accelerations were measured surface points A8-A11 and internal points A5-A9. For Experiment 1, Fig 14a shows the soil rupture process under varying peak microseismic accelerations at Specify if A3/A4 are surface or internal points. Along the slope surface, Point A8 exhibited a significantly stronger acceleration response than Point A11 (Fig 14a), while internally, Point A9 showed a higher acceleration than that at A5 (Fig 14b). With an increase in the bedrock displacement, the peak accelerations increased; in the second stage (50 mm), the maximum peak acceleration reached 0.011 g. By the third stage (80 mm), the sandy soil on the slope shoulder began to loosened, reduced the shear strength and caused the peak accelerations to decline, which indicated that the rupture propagation was approaching cessation and the slope was approaching instability. In Experiment 2 (Fig 15), throughout the three displacement stages in both the slope direction (A8–A11) and vertical direction inside the slope (A5–A7, A10), the peak microseismic accelerations at all positions, only positions near the slope toe exhibited significant changes. This suggests that bedrock faults primarily affect the rupture zone in the slope toe model, without inducing fracturing in distal slope regions on the slope.

### Slope soil pressure analysis

**Fig 16:**
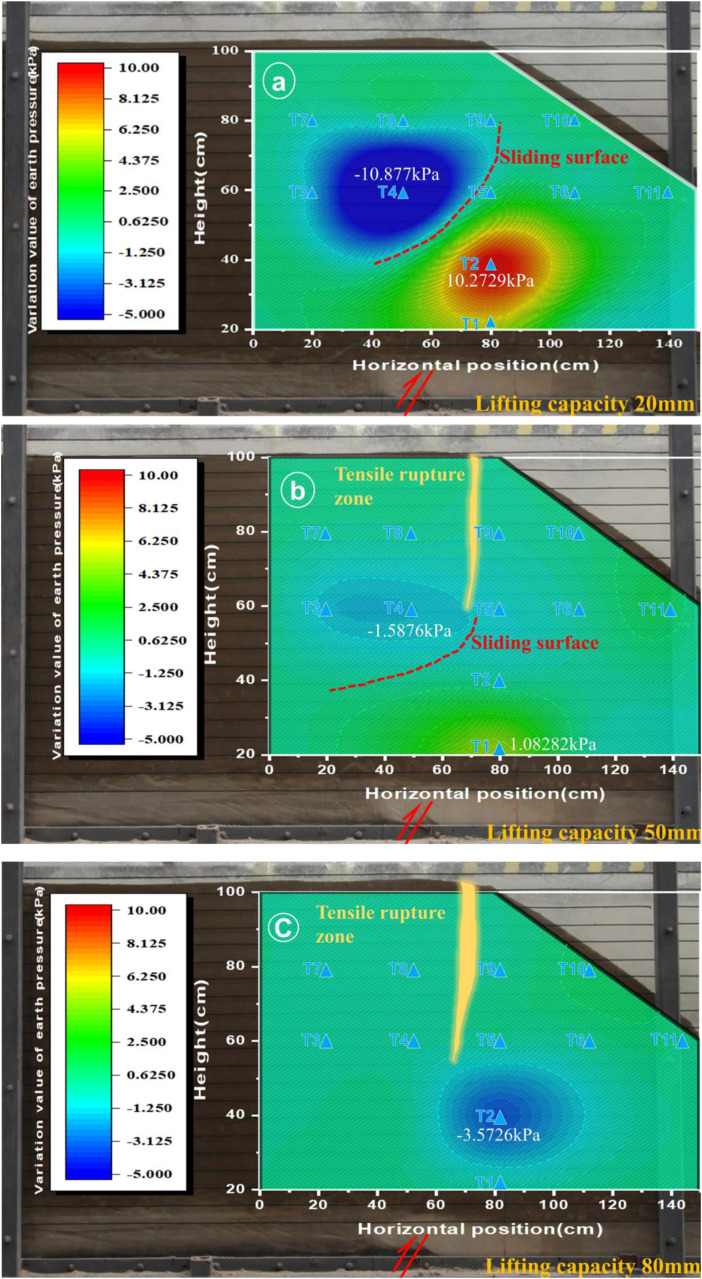
Cloud map of soil pressure changes in slope shoulder soil failure (Experiment 1).

**Fig 17:**
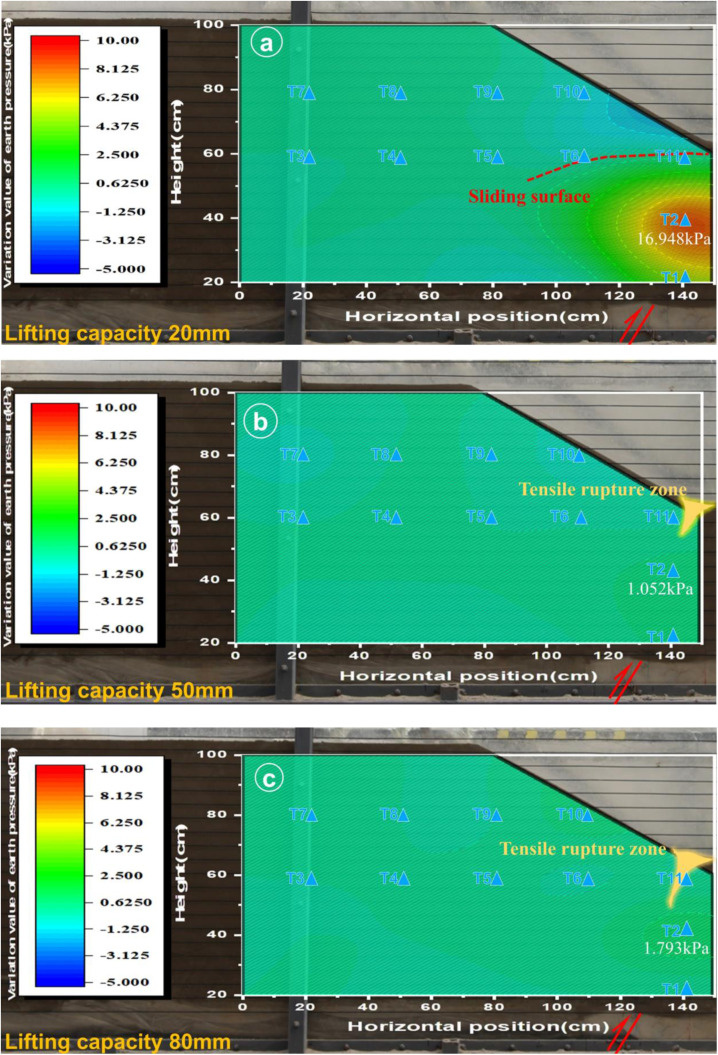
Cloud map of soil pressure changes due to soil failure at the toe of the slope (Experiment 2).

From the results shown in Figs 8–13, as well as Figs 16 and 17, it is clear that soil pressure monitoring can enables characterization of the plastic zone and rupture mechanism of the slope soil under stress fluctuations. In the initial phase, i.e., for a displacement of 20 mm, both Experiments 1 and 2 produced localized soil stress concentration due to variations in the soil pressure, leading to the accumulation of plastic zones or stress boundary zones initiating subsequent soil rupture and surface rupture development. Subsequently, in the second stage, distinct rupture zones formed at the shoulder or toe of the slope, without correlative pressure fluctuations. Moving into the third stage, the slope soil rupture zone further expanded and deepened compared to the previous stage, though minimal pressure variations in the soil pressure were observable in the corresponding graphs.

In Experiment 1, during the first stage (20 mm), the slope shoulder area experienced a compression-shear state, forming a plastic zone and minor cracks. By Stage II (50 mm), the initial cracks had elongated and propagated downward, while in the third stage (80 mm), the soil rupture zone extended further downward through the slope body. Concurrently, the tensile cracks on the slope shoulder and surface continued to widen and reach full development, signifying significant internal damage within the slope. The loosening of the internal soil led to a forward collapse of the slope, accompanied by severe tensile cracking at the shoulder, ultimately triggering global instability (Fig 16). In Experiment 2, a compression-shear zone materialized at the toe of the slope in Stage I (20 mm), nearly penetrating it. Consequently, the soil pressure monitoring readings approached null values in both Stage II (50 mm) and Stage III (80 mm), indicating that the failure zone was solely a compression-shear type at the slope toe, with no tensile failures observed (Fig 17).

### Cross-verification of microseismic responses and earth pressures

Stage I: Fracture Incubation – During initial testing (20 mm dislocation), microseismic sensors detected acceleration responses (peak 0.002−0.004g) at the slope shoulder (A8) and within the slope mass (A9), indicating localized microfracture initiation. Synchronous earth pressure monitoring (Figs 16–17) revealed: compressive-shear stress concentration at the shoulder in Test 1 forming a plastic zone with incipient cracking; and a continuous compression-shear zone at the toe in Test 2. Elastic energy accumulated in stress-concentrated areas released through micro-fracturing events, manifested by emerging microseismic signals and spatial earth pressure variations, collectively revealing initial fracture incubation conditions.

Stage II: Fracture Propagation – At 50 mm dislocation, Test 1 exhibited peak acceleration of 0.011g at the shoulder (Fig 14a) with synchronous enhancement at internal A9 (Fig 14b), marking downward tensile crack propagation. Critical earth pressure transitions occurred: abrupt reduction at Test 1 shoulder (Fig 16) due to stress release from crack penetration; whereas Test 2 toe maintained elevated pressures without significant microseismic amplification. Tensile failure (Test 1 shoulder) amplified microseismic peaks, while compression-shear failure (Test 2 toe) via soil dilation/fragmentation (constant pressure, stable micro seismicity), demonstrating failure-mode dominance over energy transfer pathways.

Stage III: Prefailure Precursors – At 80 mm dislocation, Test 1 displayed characteristic instability precursors: attenuated microseismic peaks due to reduced elastic wave transmission efficiency in loosened soil; and shoulder earth pressure approaching null values (Fig 16), confirming structural disintegration. Test 2 exhibited localized failure saturation with toe pressure stabilizing at 1–2 kPa and persistently weak microseismic response. The synchronous occurrence of microseismic decay and pressure nullification provided dual-parameter evidence for global instability (Test 1), whereas Test 2 manifested only localized compression-shear failure.

### Slope failure mechanism

**Fig 18:**
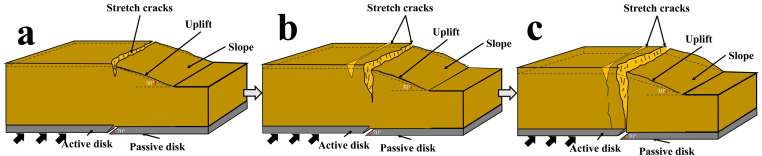
Failure mode of slope shoulder soil during reverse bedrock fault movement.

**Fig 19:**
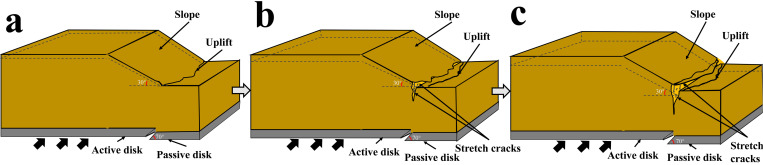
Diagram of soil failure mode at the toe of the slope during reverse bedrock fault movement.

Based on an analysis of the deformation and failure characteristics of the slope discussed above, the progressive failure process can be qualitatively divided into three stages: soil rupture incubation (Stage I), development of the soil rupture zone (Stage II), and potential landslide (Stage III). Figs 18 and 19 illustrate the failure process of sandy soil slopes influenced by reverse bedrock faults.

Stage I: The failure of the slope shoulder is initiated through upward shear surface development in the overburden at the lower part of the slope body. Concurrently, tensile cracks extend downward on the slope surface near the slope shoulder, accompanied by slight bulging deformation (Fig 18a). At the toe of the slope, passive compression-shear cracks develop, leading to a displacement characterized by the pushing of the rear sliding bodies against the front ones. Some sliding bodies are squeezed out onto the platform, accumulating at the toe and front edge of the slope, thereby initiating debris deposition (Fig 19a).

Stage II: At the slope shoulder, tensile cracks near the shoulder extend downward due to the upward shear sliding surface in the overburden, evolving into tensile rupture zones that propagate into the slope body (Fig 18b). Significant bulging deformation occurs on the slope surface. At the toe of the slope, compression-shear cracks gradually expand, enhancing toe accumulation. The compression-shear in the rupture zone generates new cracks, which advance and accumulate at the front edge of the slope (Fig 19b).

Stage III: The tensile rupture zone near the slope shoulder continues downward with bedrock dislocation, penetrating the slope body and forming a narrow-to-wide “wedge-shaped” rupture zone. This transforms the slope into a critical state with a “two-sided slope,” thereby rendering the slope unstable (Fig 18c). At the toe of the slope, passive compression-shear cracks are fully formed, resulting in a significant accumulation of soil at the toe and front edge. This failure initiates from the critical “locking segment” at the bottom (Fig 19c).

This study employs a homogeneous slope physical model (neglecting key environmental factors including rainfall infiltration and soil spatial variability) to reveal two characteristic failure modes with their instability mechanisms and associated risks through quasi-static simulation of reverse fault dislocation scenarios.

The slope-shoulder failure mode manifests through sliding surface development in lower overburden soils initiated by compression-shear mechanisms, inducing tensile rupture zones at the shoulder and bulging deformation on the slope face. This creates a new steep free face, transforming the geometry into a compound free-face geometry. Under these specific model constraints, such geometric alteration may induce secondary landslides through subsequent seismic shaking or rainfall infiltration. Conversely, the slope-toe failure mode features compression-shear fragmentation zone formation at the base, eliminating toe confinement support for the upper slope mass. Under these specific model constraints, the loss of basal constraint may trigger deep-seated progressive sliding via gravitational potential release or seismic coupling, culminating in global instability.

Physical modeling demonstrates that under strong reverse fault dislocation, the slope-shoulder failure mode presents higher acute instability potential on slope stability by directly altering geometric integrity and accelerating instability development, while the slope-toe failure mode generates latent cascading hazards.

### Verification and analysis of four earthquake-damage cases

The slope model established in this study enabled identification of key causal mechanisms and dynamic characteristics of slope instability. To comprehensively verify these findings, typical cases of fault-displacement-triggered landslides we selected representative cases for verification and analysis.

### Examples of slope shoulder damage and seismic damage

For representative seismic damage cases involving slope shoulder failure, prominent cases we analyzed, such as the Woqian landslide in Qingchuan County and the Wangjiayan landslide in Beichuan during the 2008 Ms 8.0 earthquake.

**Fig 20:**
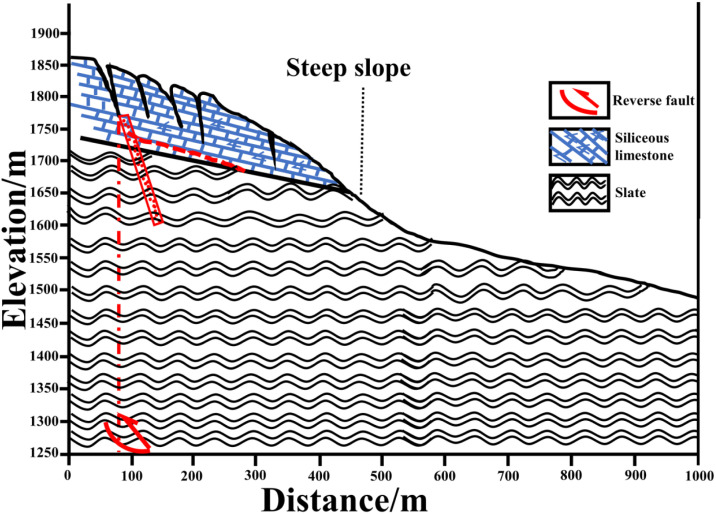
The sketch map of formation of Woqian Landslide (Source of information) [48].

The Woqian landslide occurred on the hanging wall of the Shikan Fault, exhibiting~2.31 m of strike-slip and 1.25 m of thrust components. The rear edge of the slope experienced a surface rupture zone with a maximum width of 7.9 m. This formed the primary landslide surface. The intense bedrock dislocation led to penetration of the sliding surface at the base, with developed tensile rupture zones extending hundreds of meters at the rear and side edges of the steep slope, causing the slope to loosen, crack, and disintegrate (Fig 20).

**Fig 21:**
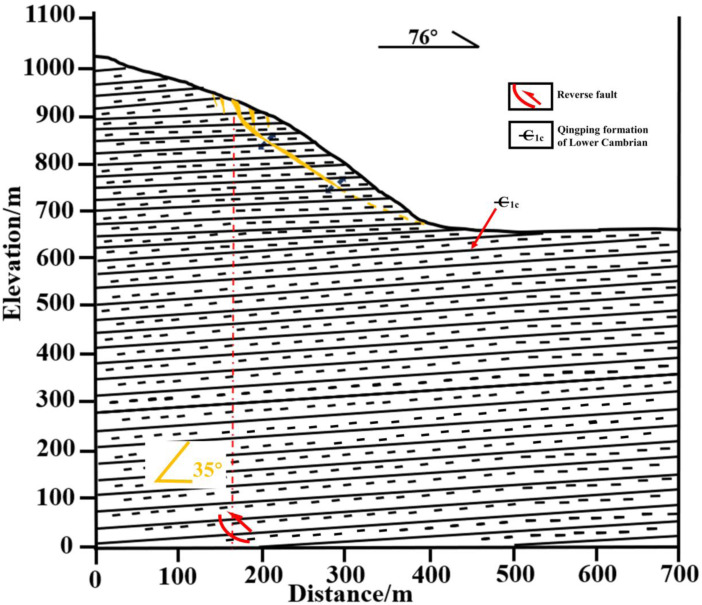
The sketch map of the formation of Wangjiayan landslide (Source of information) [48].

The Wangjiayan landslide initially loosened by seismic activity along the main bedrock fault, and resulting in a catastrophic landslide burying most of the old town and caused numerous casualties. On-site investigations revealed that rupture initiated predominantly at the slope-shoulder of the slope, extending into the mountain body and causing tensile rupture damage. The seismic activity deepened and elongated the structural planes near the top of the slope, creating the initial conditions for the landslide. Subsequent fault movements deepened the trailing edge tear surface, and under sustained seismic action, the toe of the slope could not sustain the compressive stress. This led to a complete failure of the shear-slip surface and the formation of the Wangjiayan landslide (Fig 21).

### Slope toe damage case studies

The seismic damage exhibiting slope toe failure mechanisms was examined by focusing on the Guershan No. III landslide as a representative case.

**Fig 22:**
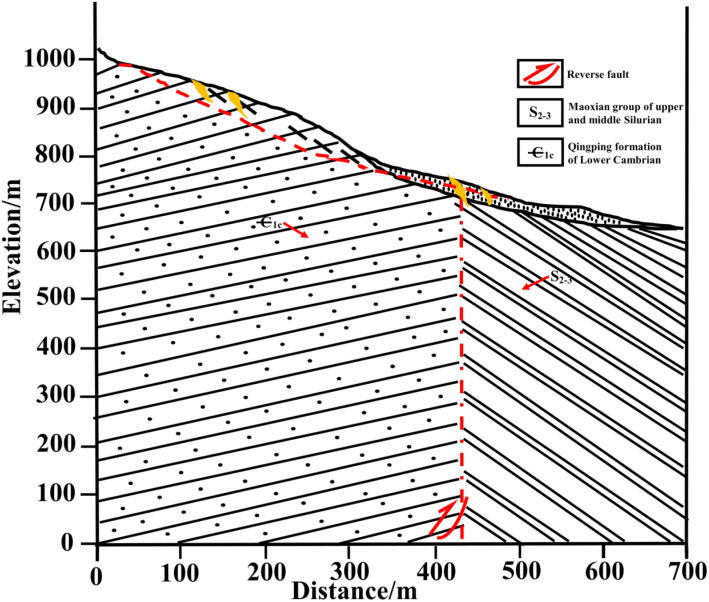
The sketch map of the formation of No.3 landslide, Guershan (Source of information) [48].

The Guershan No. III landslide, which is part of a large landslide group triggered by the 2008 Ms 8.0 earthquake, initiated when the ground motion from the Beichuan fault induced tensile fracturing throughout the slope mass in the hanging wall. Sliding surface propagated through these fissures, accelerating the movement of the slope. Despite the obstruction at the slope toe platform, a significant volume of debris accumulated due to the scale of the landslide. Rearward movement of the mass displaced some of the debris beyond the platform, depositing debris across the toe and frontal margin and front edge, resulting in debris accumulation (Fig 22).

An analysis of several earthquake damage cases revealed genetic mechanisms and dynamic characteristics consistent with those obtained in slope model tests. The predominant failure mode observed was tensile-shear, exhibiting a progressive three-stage behavior: initial slope fracture preparation, fracture zone development, and potential landslide initiation, with the hysteresis in the landslide occurrence was linked to bedrock faulting, where the fracture zone on the slope formed a sliding or free surface, entering a metastable stage before subsequent large-scale movement induced by seismic activity, rainfall, or gravitational stress. Field investigations indicated that the location of the main fracture zone on the slope corresponds to the triggering location of underlying bedrock faults, underscoring the usefulness of slope surface investigations in identifying underlying seismogenic faults.

## Conclusions

Shear and tensile ruptures induced by bedrock fault dislocations in slope bodies are a major concern in geotechnical engineering due to their potential impact on slope stability, and structural safety; a thorough analysis of these ruptures is essential for assessing slope stability and designing effective preventive measures.

This study introduced the field model test device for sandy soil slopes subjected to reverse-fault dislocations. With this device, the hazardous effects, including microseismic response, slope deformation, internal soil pressure, and macroscopic rupture characteristics, could be comprehensively examined while highlighting their implications for soil slope stability. The primary research findings are as follows:

(1) Genetic Mechanisms and Dynamic Characteristics of Reverse Fault-Displaced Sandy Soil Slopes.

The evolutionary process of reverse fault-displaced sandy soil slopes comprises three distinct phases:1) Phase I: Incubation stage of slope soil fracture initiation. 2) Phase II: Development stage of fracture zone propagation. 3) Phase III: Latent landslide formation stage.

Mechanistic analysis Shoulder failure: Tensile fracture zones and slope surface bulging deformations at the slope shoulder result from compressive-shear sliding surface formation within underlying cover soils, inducing tensile-dominated slope instability. Toe failure: Development of basal compressive-shear fracture zones eliminated lower locked-segment support, triggering shear-driven global instability.

(2) Under the controlled conditions of this experimental model (excluding key field complexities such as rainfall infiltration and soil heterogeneity), this study reveals two macroscale failure mechanisms triggered by bedrock fault dislocation.

Shoulder rupture mode: This mechanism is characterized by significant sliding surface development within the lower overburden, culminating in a transecting tensile rupture zone at the slope shoulder that traverses the slope mass. This rupture induces pronounced bulging deformation toward the free face. Microseismic monitoring and earth pressure contour maps further reveal the dynamic formation of an inverted wedge-shaped rupture zone at the shoulder, progressing toward a critical double free-face instability condition.

Toe rupture mode: This mechanism primarily manifests as a compression-shear rupture zone concentrated at the slope toe, propagating toward the foundation. Fault thrust forces drive predominantly translational slope movement predominantly translational movement. This process involves shear rupture at the toe causing material extrusion and accumulation body formation at the leading edge. This results in loss of basal confinement, thereby precipitating critical instability.

Within specific ranges of intense fault dislocation physical models (including homogeneous materials, simplified boundary conditions, exclusion of dynamic environmental factors such as rainfall or seismic aftershocks), slope shoulder failure modes exert a more significant influence on the stability of soil slopes.

(3) Model tests and on-site earthquake damage examples demonstrated a strong correlation between the position of the primary rupture zone on the slope surface (shoulder or toe) and the displacement across the bedrock fault. This zone serves not only as a geological indicator but also as a crucial criterion for identifying the location of bedrock faults.

This insight introduces a novel perspective and methodology applicable to slope engineering design, construction, and geological hazard assessment.

(4) Building upon key findings regarding slope failure modes under fault dislocation (specifically: incipient cracking susceptibility at shoulders, basal shear action at toes, and microseismic activity as instability precursors), this study yields the following practical implications: 1) Geotechnical investigations should prioritize investigations, slope shoulders should be prioritized for crack mapping and monitoring; 2) Reinforcement designs must emphasize shear reinforcement design at toe regions; 3) For critical/high-risk slopes, we recommend implementing an integrated monitoring system combining distributed optical fiber sensing (shoulder cracks); microseismic monitoring (internal damage); and toe deformation instrumentation—coupled with early-warning thresholds for dynamic risk assessment. These actionable measures translate experimental findings into enhanced risk governance capabilities for fault-zone slopes.
